# Supplementary material for: Loss of the APP regulator RHBDL4 preserves memory in an Alzheimer’s disease mouse model
Source: bioRxiv. 2024 Sep 8:2024.02.22.579698. Originally published 2024 Feb 26. Preprint. [Version 2] doi: 10.1101/2024.02.22.579698 (PMC10925189; doi:10.1101/2024.02.22.579698)
Supplement: 1 [file NIHPP2024.02.22.579698v2-supplement-1.pdf]

bioRxiv preprint doi: https://doi.org/10.1101/2024.02.22.579698; this version posted September 8, 2024. The copyright holder for this preprint (which was not certified by peer review) is the author/funder, who has granted bioRxiv a license to display the preprint in perpetuity. It is made available under aCC-BY 4.0 International license.

Supplemental Figure 1: Cognitive defects are improved in the absence of RHBDL4 expression in a different APPtg model.

A. Schematic representation of experimental design and analysis timeline for preliminary cohort of APPtg McGill-Thy1-APP mice crossed to the R4$^{-/-}$ model.

B. Spontaneous alternation performance (SAP) score from Y maze test of WT, APPtg, R4$^{-/-}$, APPtg/R4$^{-/+}$, and APPtg/R4$^{-/-}$ mice. Female data points are in purple and male in green, n=12-14 per group. Box and whisker plots represent minimum to maximum values with median center lines while blue "+" represents the mean. One-way ANOVA (p=0.008) with Dunnett's multiple comparison test, significant p-values for post hoc analysis reported.

bioRxiv preprint doi: https://doi.org/10.1101/2024.02.22.579698; this version posted September 8, 2024. The copyright holder for this preprint (which was not certified by peer review) is the author/funder, who has granted bioRxiv a license to display the preprint in perpetuity. It is made available under aCC-BY 4.0 International license.

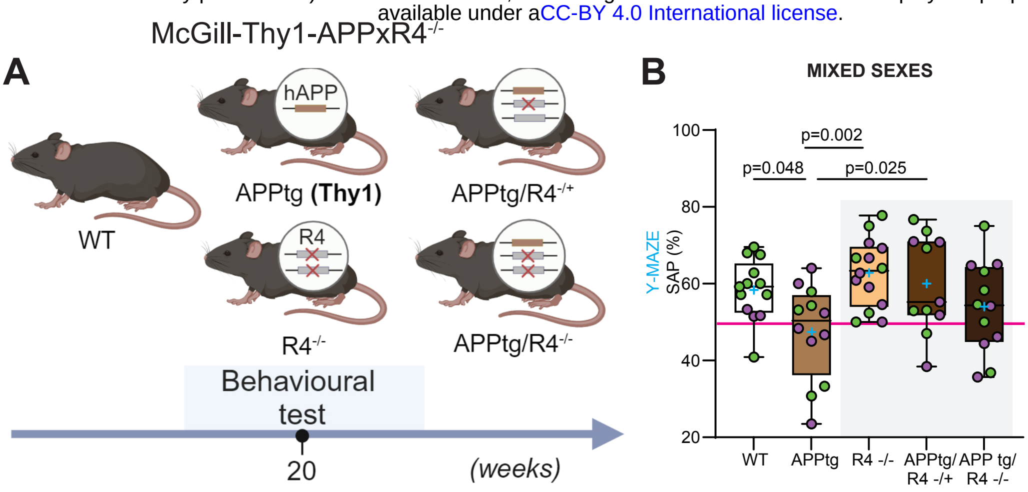

Supplemental Figure 1
